# Global patterns of healthy life expectancy in the year 2002

**DOI:** 10.1186/1471-2458-4-66

**Published:** 2004-12-24

**Authors:** Colin D Mathers, Kim Moesgaard Iburg, Joshua A Salomon, Ajay Tandon, Somnath Chatterji, Bedirhan Ustün, Christopher JL Murray

**Affiliations:** 1 Evidence and Information for Policy, World Health Organization, Avenue Appia, Geneva, Switzerland; 2 European Office of the World Health Organization, Copenhagen, Denmark; 3 Harvard School of Public Health, Harvard University, Cambridge MA, USA

## Abstract

**Background:**

Healthy life expectancy – sometimes called health-adjusted life expectancy (HALE) – is a form of health expectancy indicator that extends measures of life expectancy to account for the distribution of health states in the population. The World Health Organization reports on healthy life expectancy for 192 WHO Member States. This paper describes variation in average levels of population health across these countries and by sex for the year 2002.

**Methods:**

Mortality was analysed for 192 countries and disability from 135 causes assessed for 17 regions of the world. Health surveys in 61 countries were analyzed using new methods to improve the comparability of self-report data.

**Results:**

Healthy life expectancy at birth ranged from 40 years for males in Africa to over 70 years for females in developed countries in 2002. The equivalent "lost" healthy years ranged from 15% of total life expectancy at birth in Africa to 8–9% in developed countries.

**Conclusion:**

People living in poor countries not only face lower life expectancies than those in richer countries but also live a higher proportion of their lives in poor health.

## Background

In the *World Health Report 2000*, the World Health Organization (WHO) reported for the first time on the average levels of population health for its 191 member countries using a summary measure that combines information on mortality and morbidity [1,2]. Because substantial resources are devoted to reducing the incidence of conditions that cause ill-health but not death and to reducing their impact on people's lives, it is important to capture both fatal and non-fatal health outcomes in any such measure of population health. Healthy life expectancy – sometimes called health-adjusted life expectancy (HALE) – is a form of health expectancy indicator that extends measures of life expectancy to represent the average health in a population in terms of equivalent years of full health, taking into account the distribution of health states [3].

HALE has been calculated previously for Canada and Australia using population survey data on disability [4-6]. The United States has adopted a public health policy goal to increase the expected years of healthy life in the population and has used a type of healthy life expectancy to measure progress towards this goal [7,8]. In calculating HALE for 191 WHO Member States for 1999, we carried out an analysis of 62 representative population health surveys which revealed substantial problems with comparability of self-report health status and disability data [2]. We used health state prevalence estimates from the Global Burden of Disease 2000 project to adjust for biases in self-report data; the independent information on levels of population health provided by the health surveys was thus quite limited.

It has long been known that health expectancy estimates based on self-reported health status information are not comparable across countries due to differences in survey instruments and cultural differences in reporting of health [9,10]. The International Network on Health Expectancy (REVES) and international agencies have devoted substantial efforts to try to standardize questionnaire instruments and methods [11-13]. Though some cross-national surveys using a common self-report instrument have become available [14], standardized instruments alone do not solve comparability problems [15]. These relate more fundamentally to unmeasured differences in expectations and norms for health, so that the meaning different populations attach to the labels used for response categories in self-reported questions, such as mild, moderate or severe, can vary greatly.

Given these problems, WHO undertook a Multi-Country Survey Study on Health and Responsiveness (MCSS) in 2000 and 2001 in collaboration with Member States using a standardized health status survey instrument together with new statistical methods for adjusting biases in self-reported health [16-19]. These new data, together with comprehensive analyses of epidemiological data for all regions of the world, and new life tables for all WHO Member States, have enabled us to calculate HALE for 192 countries for 2002 in a way that improves comparability across countries. These results are reported in the *World Health Report 2004 *[20]. A previous paper has examined variations in HALE among OECD countries [21]. This paper examines the implications of the results for our understanding of global patterns of health.

## Methods

Calculation of HALE requires three inputs: life tables, prevalences of various health states, and valuations of time spent in these health states compared to full health. The WHO methods used to calculate HALE have been developed to maximise its comparability across populations. These methods are described in more detail elsewhere [22,23] and have been reviewed by an independent scientific peer review group [24]. A set of spreadsheet tools are also under development to provide full access to the inputs and calculations for country-specific HALE for 2002; these will enable users to modify inputs and to carry out sensitivity analyses for various factors. This section provides an overview of these methods and data sources and a more detailed description of adjustments for institutionalized populations.

### Life table methods

Procedures used to estimate the 2002 life tables differed for Member States depending on the data availability to assess child and adult mortality. Complete or incomplete vital registration data together with sample registration systems cover 72% of global mortality. Survey data and indirect demographic techniques provide information on levels of child and adult mortality for the remaining 28% of estimated global mortality. Separate estimates were used for the numbers and distributions of deaths due to HIV/AIDS in countries with a substantial HIV epidemic [25]. A full overview of methods used to construct life tables is given elsewhere [20,26,27].

Following an initial scientific review [24], significant improvements were implemented in both data and methods used to calculate life expectancies for WHO Member States. Recent surveys and censuses provided substantially more information on levels of child and adult mortality in Member States lacking complete death registration. This has resulted in changes in point estimates of life expectancies and reductions in uncertainty ranges for some Member States compared to previously published estimates.

### Health state prevalence data

**Figure 1:**
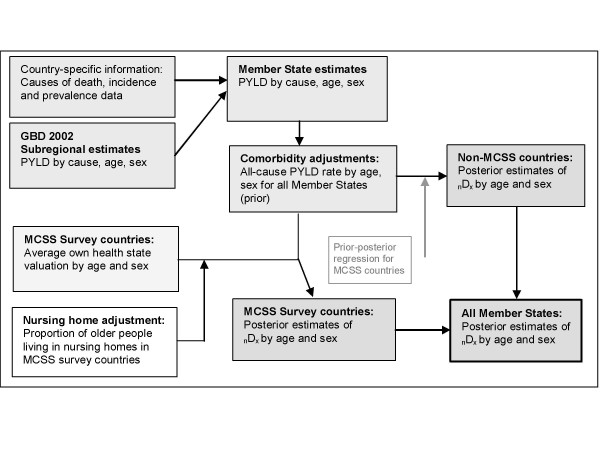
Estimation of severity-adjusted health state prevalences for calculation of HALE.

Because comparable health state prevalence data are not yet available for all countries, two sources of information were used: the Global Burden of Disease (GBD) study and the MCSS. Data from the GBD study were used to estimate severity-adjusted prevalences by age and sex for all 192 countries [23]. Secondly, data from the MCSS were used to make independent estimates of severity-adjusted prevalences by age and sex for 55 countries. Finally, 'posterior' prevalences for all countries were calculated based on the GBD-based prevalences and the survey prevalences as described below. This process is summarized in Figure 1.

The GBD revisions draw on a wide range of data sources to develop internally consistent estimates of incidence, prevalence, duration and years lived with disability (YLD), for 135 major causes, for 14 sub-regions of the world [20]. Prevalence-based YLD rates were calculated, and adjusted for co-morbidity, giving direct estimates of the severity-weighted prevalence of health states attributable to each cause [23].

Tables 1 and 2 summarize the major causes of YLD for developed and developing countries in the year 2002 as published in the *World Health Report 2004 *[20]. Neuropsychiatric conditions accounted for 42% of YLD in developed countries and nearly 30% of YLD in developing countries. Unipolar depression is the leading contributor to this burden. Other major causes of YLD include vision and hearing loss (9% in developed countries and 13% in developing countries) and injuries (nearly 12% in developing countries and 7% in developed countries). More detailed estimates of YLD by age group and cause are available for 14 subregions of the WHO Regions, and for countries grouped into high, medium and low income categories, on the WHO website at .

**Table 1:** Leading causes of disability, years lived with disability (YLD) by cause as percent of YLD from all causes, developed countries^a^, 2002

**Cause group**	**% of total YLD**	**Female to male ratio**
***I. Communicable, maternal, perinatal and nutritional conditions***	***6.6***	***1.47***
Infectious and parasitic diseases	2.4	0.94
Maternal conditions	0.9	-
Perinatal conditions	0.8	0.95
Nutritional deficiencies	2.1	1.50
***II. Noncommunicable diseases***	***86.2***	***1.12***
Malignant neoplasms	2.4	1.54
Diabetes mellitus	2.3	1.10
Neuropsychiatric conditions	41.9	1.10
*Unipolar depressive disorders*	*15.0*	*1.69*
*Bipolar disorder*	*2.2*	*0.99*
*Schizophrenia*	*2.3*	*0.94*
*Alcohol use disorders*	*6.8*	*0.24*
Alzheimer and other dementias*	*4.2*	*1.99*
*Drug use disorders*	*1.7*	*0.34*
*Other neuropsychiatric disorders*	*9.7*	*1.50*
Sense organ diseases	8.6	1.16
*Vision disorders^b^*	*3.0*	*1.44*
*Hearing loss, adult onset*	*5.7*	*1.04*
Cardiovascular diseases	6.7	0.86
Respiratory diseases	6.9	0.96
Musculoskeletal diseases	7.6	1.53
Other non-communicable diseases	9.7	1.09
***III. Injuries***	***7.2***	***0.45***
Unintentional injuries	5.8	0.48
Intentional injuries	1.4	0.31

a Developed countries includes European countries, former Soviet countries, Canada, USA, Cuba, Japan, Australia, New Zealand, Brunei Darussalam, Singapore.

b Vision disorders includes vision loss due to glaucoma, cataracts, macular degeneration and other age-related vision loss.

**Table 2:** Leading causes of disability, years lived with disability (YLD) by cause as percent of YLD from all causes, developing countries^a^, 2002

**Cause group**	**% of total YLD **	**Female to male ratio**
***I. Communicable, maternal, perinatal and nutritional conditions***	23.4	1.36
Infectious and parasitic diseases	10.9	0.95
Maternal conditions	3.8	-
Perinatal conditions	3.1	0.98
Nutritional deficiencies	4.4	1.08
***II. Noncommunicable diseases***	64.9	1.03
Malignant neoplasms	0.3	2.02
Diabetes mellitus	1.1	1.11
Neuropsychiatric conditions	29.4	1.09
*Unipolar depressive disorders*	11.1	1.47
*Bipolar disorder*	2.5	0.98
*Schizophrenia*	2.9	0.97
*Alcohol use disorders*	2.5	0.15
Alzheimer and other dementias*	1.0	1.40
*Drug use disorders*	0.8	0.28
*Other neuropsychiatric disorders*	8.6	1.37
Sense organ diseases	13.0	1.13
*Vision disorders^b^*	8.7	1.26
*Hearing loss, adult onset*	4.3	0.92
Cardiovascular diseases	3.3	0.82
Respiratory diseases	4.2	0.72
Musculoskeletal diseases	4.6	1.18
Other non-communicable diseases	8.9	0.90
***III. Injuries***	11.7	0.66
Unintentional injuries	9.7	0.75
Intentional injuries	2.0	0.32

a Developing countries includes all countries except for European countries, former Soviet countries, Canada, USA, Cuba, Japan, Australia, New Zealand, Brunei Darussalam, Singapore.

b Vision disorders includes vision loss due to glaucoma, cataracts, macular degeneration and other age-related vision loss.

Summation of prevalence YLD across all causes would result in overestimation of the total average severity-weighted health state prevalence because of comorbidity between conditions. In earlier calculations of HALE, adjustments were made for independent comorbidity assuming that the probability of having two (comorbid) conditions would equal the product of the probabilities for having each of the diseases. For the World Health Report 2003, further work was undertaken to take dependent comorbidity into account more rigorously [28]. For many diseases, the probability of having a pair of diseases is greater than the product of the probabilities for each disease, reflecting common causal pathways (for example common risk factors causing both diabetes and heart disease) and also that one disease may increase the risk of another.

Data from five large national health surveys were analysed by age and sex to estimate "dependent comorbidity factors" for pairs of conditions. There was surprising consistency in these factors across the five surveys and the results were used for all Member States to adjust for dependent comorbidity in summation of prevalence YLD across all causes [28].

### MCSS Survey estimates

The MCSS was carried out in 2000–2001. A total of 71 surveys were completed in 61 countries using face-to-face, postal and telephone interviewing modes [16]. Thirty-five of the surveys were carried out in 31 Western and Eastern European countries, 27 surveys in 22 developing countries, and the remainder in Canada, USA, Australia and New Zealand. To overcome the problem of comparability of self-report health data, the WHO survey instrument used anchoring vignettes to calibrate self-reported health for the 6 core health domains (mobility, self care, pain, affect, work and household activities, cognition) and vision. Anchoring vignettes are short descriptions that mark fixed levels of ability (e.g. people with different levels of mobility such as a paraplegic person or an athlete who runs 4 km each day) and allow us to adjust for individual variations in the use of response categories to describe the same health state [17,18,29].

We included in the analysis only those surveys that have met certain explicit criteria that reflect the quality of survey implementation with specific reference to the health vignettes. The USA postal survey was also excluded because respondents were presented the vignettes in order of severity rather than randomized as in the case of all other surveys. Sixty-two surveys met these criteria and were included in the model. Health state prevalences from the WHO Multi-country Household Survey Study were assumed to relate to calendar year 2000. Trends in prevalence YLD between 2000 and 2002 were calculated for each Member State using the GBD estimates. Aggregated across all causes, these trends were generally small. In calculating HALE for 2002, the 2000 survey results were adjusted for likely change over two years using these estimated trends.

### Health state valuations

The health state valuations used in HALE calculations represent average population assessments of the overall health levels associated with different states. They range from 0 representing a state of good or ideal health to 1 representing states equivalent to being dead. These weights do not measure average levels of well-being or quality of life associated with health states, or imply any societal value of a person in a disability or health state. Rather they characterize health decrements on a continuum starting from the societal ideal of good health.

Household surveys including a valuation module were conducted in fourteen countries: China, Colombia, Egypt, Georgia, India, Iran, Lebanon, Indonesia, Mexico, Nigeria, Singapore, Slovakia, Syria and Turkey. Data on nearly 500,000 health state valuations from over 46,000 respondents were used to develop a mapping function that captured the average relationships between levels on the six core health domains and overall health state valuations. This average global valuation function was then applied to the vignette-adjusted health domain levels for each survey respondent in order to estimate health state valuations for the calculation of HALE [30,31].

The MCSS survey samples did not include older people resident in nursing homes or other health institutions. Because these people will generally have worse health than those resident in households, adjustments were made to account for the older population who were resident in health institutions. Fifty-four national estimates of the proportion of the population aged 65 years and over who are resident in nursing homes were collected for 36 countries from national statistical publications and international statistical databases of OECD and the World Bank. These were used to estimate the percentage of the population aged 60+ years institutionalized in MCSS countries. This ranged from 3 to 5% in most OECD countries, was highest at around 7% in the Netherlands and Sweden, was substantially lower at around 0.3 to 1% in Eastern European countries, and was close to zero for developing countries. As data on the severity distribution of health states in institutionalized populations were not available, an average disability weight of 0.5 (corresponding to a health state with mobility and self-care limitations and where the person cannot carry out usual daily activities) was assumed for the institutionalized population. Sensitivity analyses showed that the resulting HALE estimates were not sensitive to the choice of this disability weight within a plausible range of variation.

### Calculation of posterior severity-weighted prevalences

Because there is potential measurement error in severity-weighted health state prevalences derived from both household surveys and epidemiological estimates, posterior estimates of prevalence for the survey countries were calculated as weighted averages of the GBD-based prevalences and the survey prevalences [23]. The relationship between the GBD-based prevalences and the posterior prevalences was estimated for the survey countries using ordinary least squares regression and the results used to adjust the GBD 2000-based prevalences for the non-survey countries. This ensured that the use of the survey data did not introduce a prevalence differential between survey and non-survey countries, and allowed the survey evidence to be indirectly taken into account in making the best possible prevalence estimates for non-survey countries.

### Calculation of HALE and uncertainty intervals

HALE was calculated using Sullivan's method [32] based on abridged country life tables and the posterior severity-weighted prevalences. Uncertainty ranges for HALE estimates were estimated using Monte Carlo simulation techniques to quantify the uncertainty in life expectancy projections, in GBD estimates for prevalence and disability severity, and in the survey-based prevalence estimates [23].

Apart from ongoing revisions to the GBD analyses of epidemiological information on diseases and injuries, the implementation of improved methods for dealing with comorbidity has resulted in a reduction in estimated proportion of healthy years of life lost at older ages compared to previous years.

Improvements in methods used for the analysis of the MCSS survey data and in the adjustment of total YLD rates for dependent comorbidity have resulted in improved estimates of the severity-adjusted prevalence of health states and a reduction in the uncertainty associated with these estimates. For these reasons, HALE estimates for 2002 are not directly comparable with those for 2000 and 2001 published in the World Health Report 2002 [33].

## Results

The survey data identified considerable variability in the use of question response categories to describe the same level on a particular health domain and a systematic tendency for people in countries with higher income per capita to use more severe response categories in rating a given anchoring vignette. Thus the self-reported prevalence of problems for a health domain in a country may be quite different from the prevalence of problems after adjustment for these response category cut-point shifts. In France, for example, 70% of survey respondents gave self-report responses of mild or greater problems on the question for the affect domain (anxiety and depression), whereas the standardized (vignette-adjusted) prevalence at all levels higher than "none" was only 33% for France. In contrast, for some countries such as Egypt, the self-report and standardized prevalences were almost the same at around 38%.

**Figure 2:**
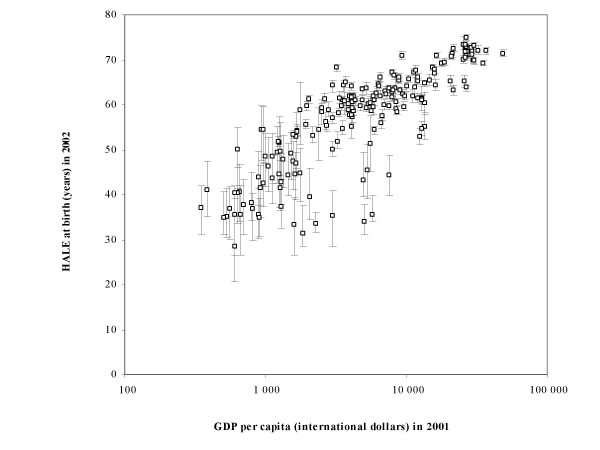
**Healthy life expectancy at birth versus Gross Domestic Product (GDP) per capita, 192 WHO Member States**. Healthy life expectancy at birth in 2002, together with 95% uncertainty ranges, versus Gross Domestic Product (GDP) per capita for 2001 in international dollars (purchasing power parity conversion), 192 WHO Member States

Figure 2 shows average HALE at birth for 192 countries, plotted against income per capita (Gross Domestic Product measured in international dollars using purchasing power parity conversion rates) on a logarithmic scale. The error bars show estimated 95% uncertainty ranges for HALE at birth. Country-specific estimates of male and female HALE and total life expectancy at birth and at age 60, together with 95% uncertainty ranges, are provided in the *World Health Report 2004 *[20].

Japanese women led the world with an estimated average HALE at birth of 77.7 years in the year 2002, 7.5 years lower than total life expectancy at birth. HALE at birth for Japanese males was 72.3 years, 5.4 years lower than for females. This male-female gap in HALE was narrower than that for total life expectancy at birth (6.9 years). After Japan, in second to seventh places, were San Marino, Sweden, Switzerland, Monaco, Iceland and Italy, with HALE at birth (males and females combined) in the range 72.7 to 73.4 years, followed by Australia and a number of other industrialized countries of Western Europe. There was a considerable range of uncertainty in the ranks for countries other than Japan, with typical 95% uncertainty ranges for HALE of around 0.5 to 2 years for developed countries. Keeping these uncertainty intervals in mind, Canada was in 11th place (72.0 years) and the USA in 29th place (69.3 years).

Other countries with reasonably high HALE in the Americas included Argentina (65.3 years), Chile (67.3 years), Costa Rica (67.2 years), Cuba (68.3 years), Mexico (65.4 years), Panama (66.2 years) and Uruguay (66.2 years). Brazil was split, with a high HALE in its southern half, and a lower one in the north. The total average was a relatively low 59.8 years, at 57.2 for males and 62.4 for females.

**Figure 3:**
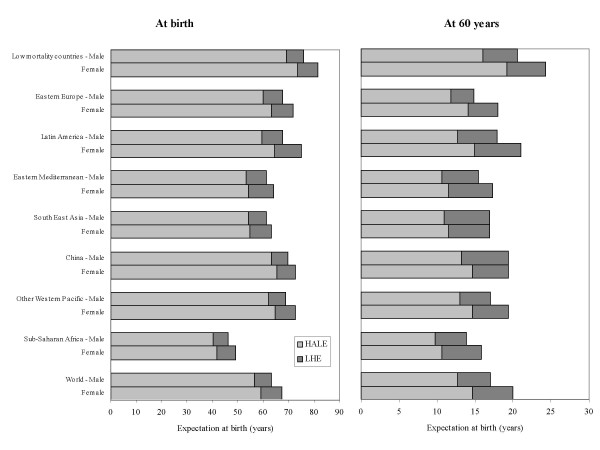
Life expectancy (LE), healthy life expectancy (HALE), and lost healthy years as per cent of total LE (LHE%), at birth and at age 60, by sex and region, 2002.

Overall, global HALE at birth in 2002 for males and females combined was 57.7 years, 7.5 years lower than total life expectancy at birth (Figure 3). In other words, poor health resulted in a loss of nearly 8 years of healthy life, on average globally. Global HALE at birth for females was only 2.7 years greater than that for males. In comparison, female life expectancy at birth was 4.2 years higher than that for males. Global HALE at age 60 was 12.7 years and 14.7 years for males and females respectively; 4.3 years lower than total life expectancy at age 60 for males and 5.3 years lower for females.

HALE at birth ranged from a low of 40 years for African males to over 70 years for females in the low mortality regions of Western Europe, North America and the Pacific (Japan, Australia, New Zealand). This reflects an almost 2-fold difference in HALE between major regional populations of the world (Figure 3). The equivalent "lost" healthy years (total life expectancy minus HALE) ranged from 15% of total life expectancy at birth in Africa to 8–9% in the European region and the Western Pacific region. The sex gap was highest for Eastern Europe and lowest in North Africa and the Middle East.

There was a similar almost two-fold variation in HALE at age 60 across the regions of the world, ranging from 19 years for women in low-mortality countries to around 10 years for men and women in sub-Saharan Africa. Males lost more healthy years of life at age 60 than females only in China and South East Asia, where life expectancy gaps at age 60 were also low compared with the more than 5-year gap in Japan (due to high female life expectancy) and in Eastern Europe (due to high adult male mortality rates).

There was an enormous difference between the world's highest HALE at birth of 77.7 years (for females in Japan) and the lowest of 27.2 years (for males in Sierra Leone) in 2002. In Sierra Leone, people both lived shorter lives (male life expectancy at birth was estimated at 32.4 years), and had higher levels of disease and disability at all ages. The probability of a male child dying before his 5th birthday was 33% in Sierra Leone, compared with less than 0.5% in Japan.

The low levels of HALE in sub-Saharan Africa reflect the additional impact of the HIV-AIDS epidemic, as well as war and conflict in some countries such as Sierra Leone. AIDS is now the leading cause of death in Sub-Saharan Africa, far surpassing the traditional deadly diseases of malaria, tuberculosis, pneumonia and diarrhoeal disease. AIDS killed 2.1 million Africans in 2002, versus 300,000 AIDS deaths in 1990 [20,34].

In Russia, HALE at birth was 64.1 for females, 4 years below the European average, but just 52.8 years for males, 9.4 years below the European average. This was one of the widest sex gaps in the world and reflects the sharp increase in adult male mortality in the 1990s. The most common explanation is the high incidence of male alcohol abuse, which led to high rates of accidents, violence and cardiovascular disease. From 1991 to 1994, the risk of premature death increased by 50% for Russian males [35]. Between 1994 and 1998, life expectancy improved for males, but has declined significantly again in the last 3 years [35-37]. Overall, HALE at birth for males in Russia and other former Soviet countries was 16 years lower than the average for males in Western Europe; the difference for females was lower at 9 years. Other Eastern European countries such as Ukraine and Belarus also had large gaps between male and female HALE at birth, as did Colombia, where male HALE at birth was nearly 9 years lower than that for females.

At the other extreme there were 12 countries where female HALE at birth was lower than male, and an additional 20 countries where it was less than 1 year higher. These included African countries greatly affected by HIV/AIDS such as Botswana, Kenya, Tanzania and Zimbabwe, but also Eastern Mediterranean countries such as Bahrain, Kuwait, Qatar and the United Arab Emirates, and Asian countries such as Afghanistan, India, Pakistan and Bangladesh. Also included were Nigeria and Haiti. In most of these countries, female life expectancy was slightly higher than male; only in Qatar, Maldives and Bangladesh was female life expectancy at birth lower than that for males. However, higher levels of disability and poor health reduced HALE for females to a greater extent than for males in these countries. At a broader regional level, sub-Saharan Africa, the Eastern Mediterranean region, and the South East Asian region all had female HALE at birth less than three years higher than male, compared with a female-male gap of 6 to 7 years in developed countries.

HALE at birth in Afghanistan was estimated at 35.5 years, the 11th lowest in the world in 2002. There was a large 95% uncertainty range around this estimate, of 27 to 44 years, reflecting the lack of population health information for that country. More health information was available for Iraq, where HALE at birth in 2002 was estimated at 50.1 years, with an uncertainty range of 47 to 54 years.

China had a healthy life expectancy well above the global average, at 64.1 years, 65.2 years for women and 63.1 for men. Other countries in the Asian region generally had lower HALE. Improving health in Viet Nam has resulted in a HALE of 61.3 years, while Thailand has not improved significantly over the past decade, with a HALE of 60.1 years in 2002. HALE in Myanmar was just 51.7 years at birth, substantially behind its South East Asian neighbors.

**Figure 4:**
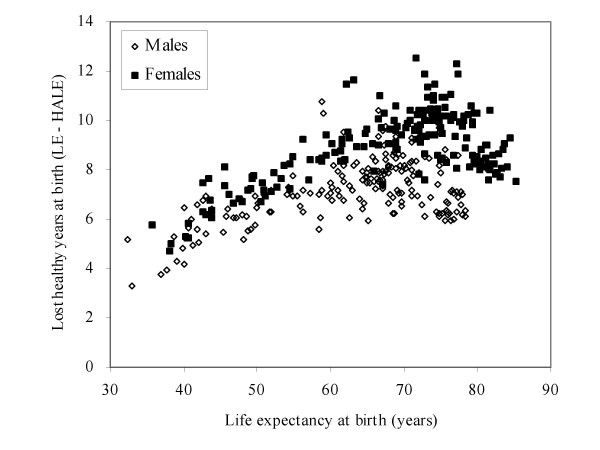
Lost healthy years at birth (LE – HALE) versus life expectancy at birth, by sex, 192 WHO Member States, 2002.

Figure 4 shows the expectation of lost healthy years at birth (LHE = LE – HALE) versus total life expectancy at birth for 192 countries. While lower life expectancies are generally associated with lower HALE, there were large variations in HALE and in LHE for any given level of life expectancy. For example, for countries with a life expectancy of 72, HALE varied from 61.1 to 64.6, a non-trivial variation.

Correspondingly, LHE varied from 7.3 to 11 years, or by up to 50%. If male and female HALE are considered separately, the range of variation increases to 59–66 at total life expectancy of 72 years. While LHE increases somewhat with increasing life expectancy up to around 70 years life expectancy, there is a flattening for males and a trend to decreasing LHE for females in the countries with longest life expectancies. Although there are higher prevalences of disabling conditions such as dementia and musculoskeletal disorders in countries with longest life expectancies, this is offset by lower levels of disability for diseases such as cardiovascular disease and chronic respiratory diseases where incidence and mortality rates are also lower.

## Discussion and conclusions

As discussed elsewhere, the new methods used in the WHO Multi-Country Survey Study offer clear evidence that self-report data on health status are not comparable, and allow adjustments that improve the comparability of the resulting health status measures [18]. Building on the findings from the MCSS, WHO is now undertaking the World Health Survey in collaboration with Member States [38]. During 2003 and the first half of 2004, 73 Member States conducted the World Health Survey, and results have now all been received by WHO. The World Health Survey results will contribute to the analysis of healthy life expectancy in future years.

Despite the fact that people live longer in the richer, more developed countries, and have greater opportunity to acquire non-fatal disabilities in older age, disability has a greater absolute (and relative) impact on healthy life expectancy in poorer countries. Separating life expectancy into equivalent years of good health and years lost to sub-optimal health thus widens rather than narrows the difference in health status between the rich and the poor countries.

Richer countries should be much more active in seeking ways to improve the health of the world's poor. WHO has been a strong advocate for efforts to increase the resources available for this purpose, and the recent report of its Commission of Macro-economics and Health concluded that the bulk of the global disease burden is the result of a relatively small set of conditions, each with an existing set of effective interventions [39]. The main problems are the funding of these interventions and access of poor populations to these interventions. The Commission estimated that the essential interventions to target these problems could be provided for a cost of around $34 per person per year.

The World Health Report 2002 included an analysis of mortality and morbidity attributable to the combined effects of 20 selected leading risk factors for 14 subregions of the world [33,40]. Globally 47% of premature mortality and 39% of total disease burden were attributable to the joint effects of the 20 selected risk factors. Removing these risk factors would lead to an estimated gain of 9.3 years (17%) in global HALE. The regional gains were between 4.4 years (6%) in the developed countries of the Western Pacific, to 16.0 years (43%) in parts of sub-Saharan Africa [41]. The World Health Report 2002 also analyzed the cost-effectiveness of a wide range of interventions to address these risks. For the first time ever, policy makers were provided not only with a summary measure of level of population health (HALE), but also with information on its determinants (diseases, injuries and major risk factors) and on the gains that could be achieved through specific intervention packages, along with an analysis of the potential improvements in healthy life expectancy for different regions of the world that could be achieved through reduction or elimination of exposure to 20 major global risk factors [33].

The regular assessment of levels of population health is a key input to the public policy process, and comparable measurement of population health levels creates possibilities of investigating broad determinants at national and cross-national level. Mortality indicators are not adequate for this purpose, given the considerable policy interest for many populations as to whether – and to what extent – gains in life expectancy have been accompanied by improvements in non-fatal health status [9,42-45]. The trend to flattening or decreasing LHE for countries with longest life expectancies, noted above, provides the first cross-population comparable evidence that compression of morbidity may be occurring in low mortality countries, although this evidence is cross-sectional rather than longitudinal.

Comparability is fundamental to the use of survey results for development of evidence for health policy but has been under-emphasized to date in instrument development. We believe that the new methods used in the WHO Multi-country Household Survey Study and the World Health Survey have increased the comparability of self-report data across countries and provide the first steps towards the consistent and comparable measurement of population health across the world. Final confirmation that compression of morbidity is occuring in low mortality populations awaits longitudinal cross-population comparable data for specific populations: this is now achievable.

## Competing interests

The author(s) declare that they have no competing interests.

## Authors' contributions

CDM, CJLM and JAS developed the methods for calculation of HALE. CJLM, JAS and AT developed the methods and carried out the calculations for the measurement of health states and health state valuations in the MCSS, with contributions from the other authors. CJLM, JAS, SC and BU developed the MCSS survey instrument, and SC and BU coordinated the implementation and analysis of the surveys. KMI, CDM and JAS carried out analyses and calculations for the computation of HALE for the year 2002 for 192 WHO Member States, with contributions from CJLM and the other authors. CDM drafted the paper, with substantial contributions from other authors.

## Pre-publication history

The pre-publication history for this paper can be accessed here:


